# Nexus between inflation, income inequality, and economic growth in Ethiopia

**DOI:** 10.1371/journal.pone.0294454

**Published:** 2023-11-16

**Authors:** Abdurhman Kedir Ali, Dagmawe Menelek Asfaw

**Affiliations:** 1 Department of Economics, College of Business and Economics, Samara University, Samara, Ethiopia; 2 Department of Economics, University of Gondar, College of Business and Economics, Gondar, Ethiopia; Universiti Malaysia Sabah, MALAYSIA

## Abstract

The relationship between inflation, income inequality, and economic growth is a subject of intense debate among economic researchers and policymakers. This study aims to analyze this relationship in Ethiopia using advanced statistical techniques such as VEC (vector error correction) model with Granger causality, and Johansen’s cointegrated. The study covers the period from 1980 to 2022 and includes pre and post-estimation diagnosis tests to ensure the accuracy of the model. The results indicate the presence of a long-run relationship among inflation, income inequality, and economic growth, as confirmed by Johansen’s cointegrated test. Additionally, the vector error correction model shows a strong long-run relationship between economic growth, income inequality, and inflation. In the short run, there is a significant association between income inequality and economic growth, as well as between inflation and economic growth. The Granger causality test reveals a bidirectional causality between economic growth and income inequality and between economic growth and inflation. However, there is a unidirectional causality from inflation to income inequality. Based on these findings, it is suggested that the government should implement various strategies and policies, including redistribution policies, social safety nets, promoting inclusive economic growth, coordinating effective monetary and fiscal policies, implementing progressive taxation, and reforming the labor market.

## Introduction

Income inequality pertains to the uneven allocation of income across a given society and measure in term of Gini coefficient. On the other hand, income inequality can have profound effects on both inflation and economic growth. High levels of income inequality may contribute to social and economic instability, as marginalized groups struggle to access resources and opportunities [1]. This can have repercussions on inflation, as limited access to necessities and increased financial strain can lead to social unrest and pressure to increase wages. Additionally, income inequality can impede economic growth by restricting market participation, hindering human capital development, and creating social divisions that undermine productive economic activities [2,3].

Inflation is an economic concept that refers to the sustained increase in the general price level of goods and services in an economy over a period of time and it can be measure in term of consumer price index (CPI). It is often measured by the inflation rate, which indicates the percentage change in prices over a specific period, typically a year. Inflation erodes the purchasing power of money, as it reduces the amount of goods and services that can be purchased with a given amount of currency.

Moderate inflation can be considered a sign of a healthy economy. This indicates that there is sufficient demand for goods and services, which encourages businesses to invest and expand, leading to economic growth [4]. High inflation rates can negatively affect economic growth. When inflation rises too quickly, it erodes consumers’ purchasing power and reduces their ability to spend. This can lead to decreased consumer confidence, lower investments, and slower economic growth. Inflation can exacerbate income inequality by increasing prices for essential goods and services and eroding the purchasing power of low-income individuals, which leads to spending a larger proportion of their income on necessities. This can lead to an escalation of income inequality by further widening the disparity between the wealthy and the less affluent [3,5].

Economic growth entails a rise in the production and consumption of goods and services over time and it can also measure in Gross Domestic Product (GDP). Generally, sustained economic growth is beneficial as it can improve living standards and provide opportunities for employment and income generation. However, the economic growth benefits may not be equally distributed, resulting in income inequality. In particular, certain sectors or regions experience more significant growth than others do. It is essential to assess whether economic growth in Ethiopia is inclusive and whether it has effectively addressed income inequality [6,7]. When an economy is rising rapidly, demand for goods and services can outpace supply, leading to higher prices/inflation [7].

Numerous studies investigate the relationship between inflation and economic growth, revealing a diverse range of findings. The studies by [8–13] finds a negative relation between inflation and economic growth. They argue that high inflation rates reduce investment and distort consumption patterns, hindering economic growth. However, contrasting results are provided by other researchers, such as [6,9], who claim that moderate inflation rates can boost economic growth by stimulating investment and reducing real wages.

Income inequality has been a growing concern in Ethiopia, warranting attention to its relationship with economic growth. Evidence suggests that high income inequality can hinder economic growth through multiple channels. For instance [14–17], argue that severe income inequality might lead to political instability, reduced human capital development, and hindered social cohesion, all of which are detrimental to economic growth. Nevertheless, there are scholars, like Nemati and Raisi [18], who suggest that a certain degree of income inequality can spur economic growth by incentivizing investment and innovation.

The impact of inflation on income inequality is another area of research interest. Rising inflation rates may disproportionately affect the poor due to their limited access to financial services resulting in lower income and higher relative prices of essential goods [7,13,19–21], find empirical evidence indicating that inflation exacerbates income inequality, widening the welfare gap between the rich and the poor. However, further research is needed to provide a comprehensive understanding of the nuanced relationship between inflation and income inequality in the Ethiopian context.

For policymakers and economists in Ethiopia, it is extremely important to prioritize sustainable and inclusive economic development. Despite facing both domestic and external challenges, the Ethiopian economy experienced consistent growth, with a 6.4 percent increase in 2021/22 compared to 6.3 percent the previous year. This growth has contributed to a steady rise in per capita income, which reached USD 1,218 in 2021/22. Additionally, the investment-to-GDP ratio stands at 25.3 percent, while the domestic savings-to-GDP ratio is 15.3 percent [22].

Inflation has been consistently above the single-digit target in the last four years. In the 2021/22 fiscal year, the average annual headline inflation reached 33.8%, compared to 20.2% in 2020/21, mainly due to increased inflation rates in both food and non-food sectors. This inflation rate was more than double the average inflation rate in sub-Saharan Africa, which stood at 14.47% in 2021/22. However, it is expected that inflation will gradually decrease in the sub-Saharan region in the coming years [23].

Similarly, following a period of reduced inequality from 2004 to 2010 (with a decrease of 6.2 percentage points), recent trends suggest a renewed widening of the income gap. The distribution of income remains uneven, with the bottom 10% of the population possessing only 4% of the income. Additionally, the Gini coefficient index reached 54.5% in 2021 [15]. This was also much higher than sub-Saharan income inequality (39.99%) [23].

Different macroeconomic policies have been launched in Ethiopia to achieve stable macroeconomic growth, such as Growth and Transformation Plan Two (GTP II); which aims to maintain macroeconomic stability by enhancing export competitiveness by creating a conducive environment. In addition, the 2030 Agenda for Sustainable Development and its 17 Sustainable Development Goals (SDGs) emphasize the need to address broad inequalities and inflation [24]. However, its objective has not been achieved specifically for inflation and income inequality [13].

Therefore, the nexus between economic growth, income inequality, and inflation in Ethiopia is a many-sided issue that requires comprehensive analysis. Understanding the dynamics and interdependencies between these variables is crucial for formulating effective economic policies that promote sustainable development and address social disparities. However, the specific nature and magnitude of the relationship between inflation, income inequality, and economic growth in Ethiopia remain largely unexplored.

The relationship between economic growth, inflation, and income inequality has been the subject of considerable debate among economists and policymakers. Thus, different studies have been conducted on these issues. One such study is "Inflation, Income Inequality, and Economic Growth in Ethiopia" by Yue [8]. This study utilized time series data spanning from 1980 to 2002 to examine the relationships between inflation, income inequality, and economic growth in both the long and short term. The findings of the study indicate that inflation has a detrimental impact on economic growth, whereas income inequality has a favorable effect on economic growth.

Another study is "The Nexus between Economic Growth, Inflation, and Income Inequality Economic Growth in Pakistan: An ARDL Approach" by Ali [25]. The study used data from 1972 to 2007 to investigate the long- and short-run relationships between inflation, income inequality, and economic growth. The study found that the growth-increasing impact of income inequality in Pakistan in the long run and inflation has a positive effect on the economy in the short run. A study " Income Distribution, Inflation, and Growth: A Cross-Country Study" by Li and Zou [21]. We used cross-country panel data on income distribution to explore the impact of inflation on income distribution and economic growth. The study finds that inflation worsens income distribution and reduces the rate of economic growth.

In general, some studies also found negative long-run and short-run relationships between inflation income inequality, and economic growth across countries [5,11,15,26–31] and …); however, other studies also investigated the positive long-run and short-run dynamics between inflation income inequality and economic growth [18,32–34] and …. Previous investigations have not fully explored the research gap that persists in Ethiopia. Specifically, there is a lack of research that examines the association between inflation, income inequality, and economic growth together. This gap exists because Ethiopia is an emerging country with unique economic characteristics and challenges. To address this, it is important to conduct specific research in Ethiopia. Additionally, policymakers would benefit from a comprehensive analysis of the macroeconomic policy implications of this relationship in order to design more effective strategies for inclusive growth. Investigating the long-term dynamics could also provide insight into critical levels of income inequality or inflation that significantly affect economic growth. However, previous literature has not adequately focused on the policy implications of this nexus in Ethiopia. Furthermore, previous studies were limited to utilized the application of the VEC model, which allows for the examination of simultaneous interactions between multiple variables, has been limited in existing research. Additionally, there is insufficient understanding of the causal direction between income inequality, inflation, and economic growth in Ethiopia. To address these gaps, this study aims to analyze the nexus between inflation, income inequality, and economic growth in Ethiopia using VEC model, by organizing by four section namely: introduction, methodology, data analysis and conclusion & recommendation.

## Methodology of the study

### Research design

This research study utilized a quantitative analytical approach.

### Data source and scope

The study utilized time series data spanning 42 years, from 1980 to 2022, sourced from various secondary databases basically from the World Development Indicator database. For triangulation purpose, we have to use: International Monetary Fund database, and the National Bank of Ethiopia. Additionally, data from the World Bank’s World Development Indicators and National Bank of Ethiopia, as well as the Ethiopia Statistical Survey and World Economic Outlook online databases, were also used. Data from international organizations were collected from their respective databases, while information from local authorities was obtained through direct visits to their offices.

### Method of data analysis

The study utilized both descriptive and econometric techniques to evaluate the relationship and patterns between economic growth, income inequality, and inflation rate over the study period. Descriptive statistical tools like trend graphs were employed to analyze the trends. In terms of the econometric model vector error correction models (VECM).

### Pre/Post estimation diagnosis test

Pre-estimation test was conducted, such as a stationarity test using both the Augmented Dickey-Fuller and PP tests and by using Akaiki Information Criteria determined the maximum lag selection. Post-estimation tests such as stability, normality, heteroskedasticity, and serial correlation under time-series analysis were conducted in this study.

### Johansen co-integration test

To determine the rank of the long-run matrix and hence the number of co-integrating vectors, the two likelihood ratio tests (the trace), and the maximal eigenvalue (λ maximum) statistics are used [35]. For both test statistics, the initial Johansen test is a test of the null hypothesis of no co-integration against the alternative of cointegration.

### Granger causality test

One of the first, and undeniable, maxims that every econometrician or statistician is taught is that “correlation does not imply causality.” Correlation or covariance is a symmetric, bivariate relationship; cov (x, y) = cov (y, x). We cannot, in general, infer anything about the existence or direction of causality between x and y by observing non-zero covariance. Even if our statistical analysis is successful in establishing that the covariance is highly unlikely to have occurred by chance, such a relationship could occur because x causes y, because y causes x, because each causes the other, or because x and y are responding to some third variable without any causal relationship between them.

The formal definition of Granger causality asks whether past values of x aid in the prediction of, conditional on having already accounted for the effects on of past values of y. If they do, then x is said to Granger cause y. The VEC is a natural framework for examining Granger causality. From the two variable system in equations, if x Granger causes y, then some or all of the lagged x values have non-zero effects. Testing for Granger causality amounts to testing the joint blocks of coefficients and, to see if they are zero.

The null hypothesis X does not Granger cause Y in this VECM is

$$H_{0}:\beta_{yx1=}\beta_{yx2=}\ldots =\beta_{yxp}=0,\;$$

The null hypothesis Y does not Granger cause X in this VECM is

$$H_{0}:\beta_{xy1=}\beta_{xy2=}\ldots =\beta_{xyp}=0,\;$$

According to Granger [36], the existence of co-integration between X and Y must be evaluated before performing a causality test. If a co-integrating relationship is identified, then causality must exist in at least one direction. Causality can be unidirectional, that is, it can run only from X to Y. On the other hands, when causality runs from one variable to the other and, in turn, runs from that variable to the other, then the causality that exists is called feedback effects. To summarize, we must be very careful in interpreting the result of Granger causality tests to reflect true causality in any non-econometric sense. Only if we can rule out the possibility of the future causing the present and strictly immediate causal effects can we confidently think of “Granger causality” as “causality.

### Econometric model specifications

#### The Vector Error Correction Model (VECM) model specification

According to Mundell [37], The level of non-stationarity in time-series variables makes the results obtained from the VAR model unreliable and misleading. It is important to properly differentiate these variables in the VAR model to avoid model misspecification, especially when the level variables exhibit a long-term relationship [38]. In such cases, it is recommended to further investigate the VAR model in vector error correction form. However, there is an exception when two or more I (1) variables are co-integrated, meaning there exists a linear combination of these non-stationary variables that are stationary. This indicates a long-run relationship between the variables, which aligns with economic theory that suggests the existence of such equilibrium relationships. Moreover, when stationary variables have a long-run relationship, it affects the short-term behavior of the I (1) variables as there must be a mechanism that drives them toward their long-run equilibrium relationship. This mechanism is captured by an error correction mechanism where the equilibrium error is instrumental in modeling the short-term dynamics of the series [39].

In addition, the vector error correction model (VECM) accounts for the possibility of all variables being endogenous [40]. Each variable, expressed in its first difference, is designed to respond to changes in other variables and deviations from the long-run equilibrium path. If there are co-integrated variables, the VECM representation can be written according to the Granger representation theorem. To encompass both short- and long-term relationships in the model, the study employs the VECM, which can be specified as follows:

$$\begin{array}{c} \Delta Y_{t}=\psi \Omega +\Pi y_{t-1}+\beta_{1}y_{t-1}+\beta_{2}y_{t-2}+\cdots +\beta_{p}Y_{t-p}+\varepsilon_{t}\Delta Y_{t}\\ =\psi \Omega +\Pi y_{t-1}+\sum_{i=1}^{p}\beta_{i}\Delta y_{t-i}+\varepsilon_{t} \end{array}$$

where Δ*Y*_*t*_ represents the first differences of the variables $\beta_{i}=-\sum_{j=i+1}^{p}\alpha_{i}$ is the (nxn) coefficient matrix in the error correction term (which contains short-run parameters), and $\pi =\sum_{i=1}^{p}\alpha_{i-1}$ is the (nxn) matrix of long-run responses, which contains information about long-run relationships. Furthermore, the error terms are assumed to be Gaussian or well-behaved [39]. The vector error correction model (VECM) includes cointegration relations in its specification, which constrain the endogenous variables to move towards their cointegrating relationship in the long run, while also accounting for short-run adjustment dynamics [39].

In this study, the tri-variate vector error correction model (VECM) is specified as

$$\Delta {lnCPI}_{t}=\alpha_{11}+\sum_{i=1}^{\rho }\beta_{1i}{\Delta lnCPI}_{t-i}+\sum_{i=1}^{\rho }\delta_{1i}{\Delta lnGINI}_{t-i}+\sum_{i=1}^{\rho }\gamma_{1i}{l\Delta nRGDP}_{t-i}+\Omega_{11}{ECT}_{t-1}{+\varepsilon }_{1t}$$

$${\Delta lnGINI}_{t}=\alpha_{21}+\sum_{i=1}^{\rho }\beta_{2i}{\Delta lnCPI}_{t-i}+\sum_{i=1}^{\rho }\delta_{2i}{\Delta lnGINI}_{t-i}+\sum_{i=1}^{\rho }\gamma_{2i}\Delta {lnRGDP}_{t-i}+\Omega_{21}{ECT}_{t-1}+\varepsilon_{2t}$$

$${\Delta lnRGDP}_{t}=\alpha_{31}+\sum_{i=1}^{\rho }\beta_{3i}\Delta {lnCPI}_{t-i}+\sum_{i=1}^{\rho }\delta_{3i}\Delta {lnGINI}_{t-i}+\sum_{i=1}^{\rho }\gamma_{3i}{\Delta lnRGDP}_{t-i}+\Omega_{31}{ECT}_{t-1}+\varepsilon_{3t}$$

where the variables are already defined; Δ denotes the difference operator; *ECT*_*t*−1_ is the one-period lagged error correction term; and *ε*_1*t*_; *ε*_2*t*_; *ε*_3*t*_ are error terms with zero mean and constant variance. In this specification, *α*′*s*; *β*′*s*; *δ*′*s*; and *γ*′*s* s are unknown coefficients, and the parameters of the *ECT*_*t*−1_ (Ω_11_, Ω_21_, *and* Ω_31_) denote the speed of adjustment in case of deviations from the long-run equilibrium relationship [39].

**Consumer Price Index (***lnCPI*_*t*_**)**: in Ethiopian Consumer Price Index (CPI) measures fluctuations in the prices of the goods and services that households typically consume. The Consumer Price Index (CPI) is a measure of the average change over time in the prices paid by consumers for a market basket of consumer goods and services. The CPI is calculated by selecting a representative sample of goods and services that are typically purchased by urban consumers. This sample, known as the market basket, includes items such as food, housing, transportation, healthcare, education, and recreation. The prices of these items are collected regularly from thousands of retail outlets, service establishments, and other providers across different geographic areas.

**Income Inequality** (*lnGINI*_*t*_): The level of income inequality can be assessed using either the GINI coefficient or quintile ratio. It ranges from zero to one, with zero denoting complete equality (where every household has the same income) and one indicating extreme inequality (where a single household possesses all the income within society). The Gini coefficient quantifies the average income disparity between all pairs of individuals, dividing it by the average income. This coefficient is derived from the Lorenz curve, which depicts the cumulative percentage of income earned by each cumulative population share.

**Real Gross domestic product (***lnRGDP*_*t*_): Gross Domestic Product (GDP) is an indicator that takes into account price fluctuations and provides a comprehensive measure of an economy’s size. It represents the total value of all final goods and services produced within a country during a specific period. The determination of GDP is influenced by both the quantity of goods and services produced, known as volume, as well as their corresponding prices. To assess the overall economic performance, Real GDP (RGDP) is utilized.

#### Theoretical framework

**Fig 1:**
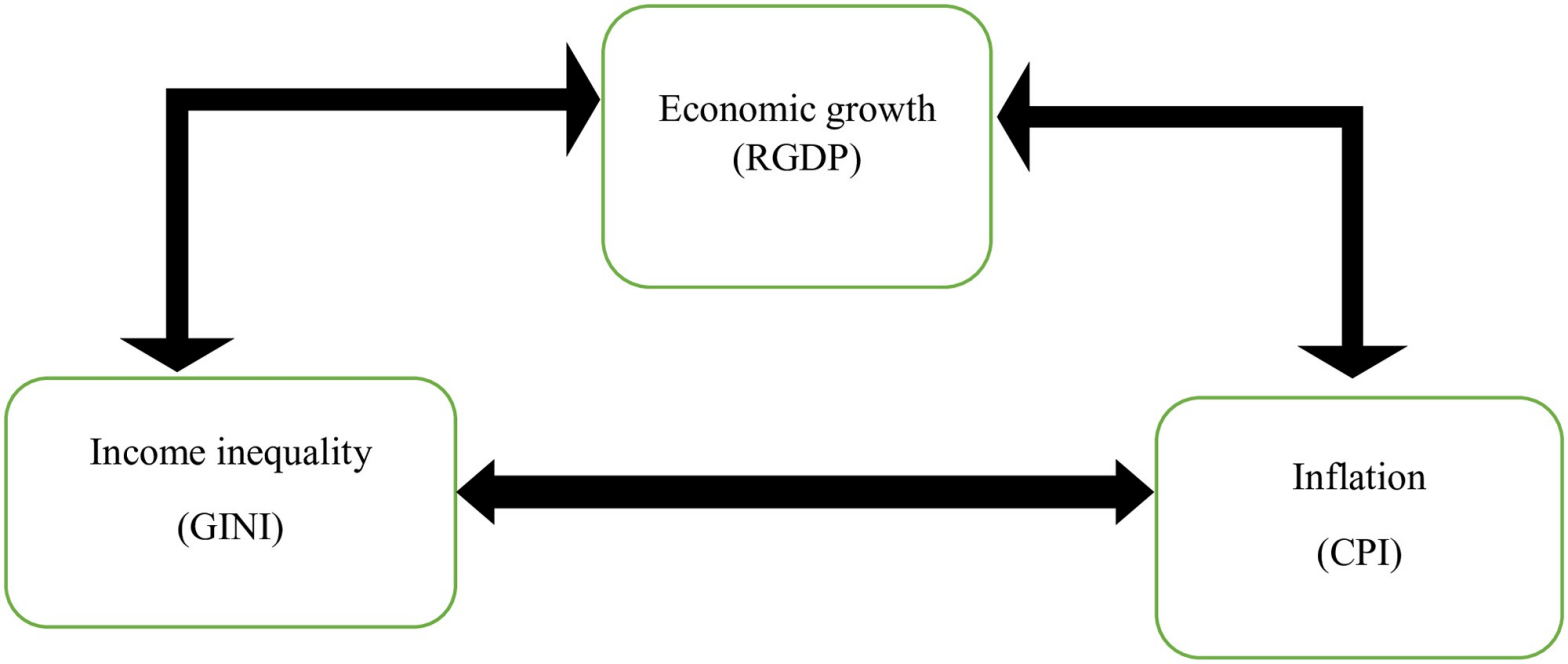
Conceptual framework adapted from different literature, 2023.

The relationship between inflation, income inequality, and economic growth has been a topic of extensive research and debate among economists. Understanding this nexus is crucial for policymakers as it helps in formulating effective strategies to achieve sustainable economic development. This theoretical framework aims to analyze the interconnections between inflation, income inequality, and economic growth specifically in the context of Ethiopia (see Fig 1).

Inflation refers to the sustained increase in the general price level of goods and services over time. It is typically measured by the consumer price index (CPI). Income inequality refers to the unequal distribution of income among individuals or households within a society. It is often measured using indicators such as the Gini coefficient. Economic growth refers to an increase in the production of goods and services within an economy over time. It is commonly measured by changes in real GDP (Gross Domestic Product). The relationship between inflation and economic growth is a subject of ongoing debate among economists. The traditional view suggests that high inflation is detrimental to economic growth as it reduces real incomes, distorts price signals, and increases uncertainty. High inflation can also lead to a misallocation of resources and hinder productive investments. However, some economists argue that moderate inflation can have a positive impact on economic growth by stimulating consumption and investment.

The relationship between income inequality and economic growth is also a topic of extensive research. The traditional view suggests that high levels of income inequality can hinder economic growth by limiting access to education, healthcare, and productive resources for a significant portion of the population. Inequality can also lead to social unrest and political instability, which can negatively impact investment and economic productivity.

High levels of inflation can exacerbate income inequality by eroding the purchasing power of low-income households disproportionately. Inflation can also hinder long-term investments in human capital development and infrastructure. Conversely, income inequality can contribute to inflationary pressures by limiting access to productive resources and opportunities for a significant portion of the population. Unequal distribution of income can lead to excessive consumption by the wealthy, which can drive up prices and create inflationary pressures. In conclusion, understanding the nexus between inflation, income inequality, and economic growth is crucial for policymakers in Ethiopia. High levels of inflation can hinder economic growth and exacerbate income inequality, while income inequality can contribute to inflationary pressures. Achieving sustainable economic development requires a comprehensive policy approach that addresses both inflation and income inequality simultaneously.

#### Chapter four: Results and discussion

This section discusses the results of vector autoregressive (VAR) and vector error correction models (VECM), including descriptive and empirical estimation results. After the estimation and discussion, the outcomes of the diagnostic test verifying the suitability of the model were provided.

#### Pre/Post estimation diagnosis test

Before proceeding to the discussion, a pre-estimation test was conducted, such as a stationarity test using both the Augmented Dickey-Fuller and PP tests and by using Akaiki Information Criteria determined the maximum lag selection. The stationarity test results indicated all variable were not stationary at level, however after taking the first difference, all variables exhibited stationarity. And also based on Akaiki Information Criteria the model’s maximum lag was one.

Post-estimation tests such as stability, normality, heteroskedasticity, and serial correlation under time-series analysis were conducted in this study. The results revealed no evidence of serial correlation, a normally distributed residual, a stable modal, and no evidence of heteroscedasticity.

### Econometric analysis

#### Cointegrated test

The dynamic multivariate VECM model aims to capture the linear interdependence between a group of variables by treating them equally. VECM models are specifically designed to depict short-term dynamic imbalances among these variables and can be utilized for analyzing the dynamics of inflation, economic growth, and income inequality relationships. To establish long-term connections, cointegrating vectors were assessed through Johansen’s cointegration method.

Determining if the variables are co-integrated in the same order, specifically order one I (1), after conducting unit root tests and determining the optimal lag duration, is of utmost importance. In the case that these variables are indeed cointegrated, they may exhibit a long-term relationship provided the series are integrated in the same order. The precise quantity of cointegrated relations is established through the utilization of the Johansen integration approach, which serves as the basis for model design and inference procedures [41]. In addition, it is crucial to analyze numerous co-integrating vectors.

Based on the Johansen approach to the cointegrated test, the rank test values for both the trace test and the maximum eigenvalue test were utilized to determine the number of cointegrating vectors in this model. The findings of these tests are presented in Tables 1 and 2. The table demonstrates that the null hypothesis of no cointegrating vector is rejected at a 5 percent critical value as indicated by the trace test statistics. However, the presence of three or fewer cointegrating equations cannot be dismissed at the conventional significance level. Therefore, based on the trace test, we can conclude that there are three cointegrating relationships in the VECM model. Moreover, the maximum eigenvalue test confirms the existence of three cointegrating vectors. Thus, according to the Johansen cointegrated test, there are three cointegrated vectors in the model, thereby confirming the long-run relationship between these macroeconomic variables.

**Table 1:** Johansen’s cointegrated tests by maximum eigenvalue test.

Hypothesized number of CE(s)	Eigenvalue	max Eigen statistic	5 percent critical value
None*	0.54321	39.9845	30.983
At most 1*	0.43693	31.7532	27.354
At most 2*	0.35687	23.5735	21.542
At most 3	0.15874	18.2364	19.456

Trace test indicates 3 cointegrating eqn(s) at the 0.05 level.

* denotes rejection of the hypothesis at the 0.05 level.

**MacKinnon-Haug-Michelis (1999) p-values.

**Table 2:** Johansen’s cointegrated tests by maximum trace statistic.

Hypothesized number of CE(s)	Eigenvalue	Trace statistic	5 percent critical value
None*	0.54321	89.4405	80.877
At most 1*	0.43693	81.2092	77.248
At most 2*	0.35687	73.0295	71.436
At most 3	0.15874	67.6924	69.35

Trace test indicates 3 cointegrating eqn(s) at the 0.05 level.

* denotes rejection of the hypothesis at the 0.05 level.

**MacKinnon-Haug-Michelis (1999) p-values.

### Vector Error Correction Model (VECM)

The Johansen cointegrated test confirms the long-term relationship between variables in the model. It checks for cointegration, which is the presence of a stable connection between variables. To analyze nonstationary series, the vector error correction (VEC) model, a reduced VAR model, is used for known co-integrated series. The VEC model allows for short-term adjustments and limits the long-term behavior of endogenous variables, ensuring that their connections converge. The VEC model can be represented in simplified VAR form. Any short-term variations or deviations from the long-term equilibrium are corrected using the VECM.

### Long-run analysis

The Johansen cointegration test reveals that our model comprises three cointegrating vectors. If the existence of cointegration among the variables is validated, we can determine their long-term equilibrium relationships. To analyze the connection between inflation, income inequality, and economic growth, it is important to consider the three cointegrating vectors, which are indicated by trace statistics and the maximum eigenvalue.

This statement presents the long-run equation for economic growth in Ethiopia, which connects real economic growth to inflation and income inequality. It forms the basis for the long-term analysis. The findings demonstrate that economic growth in Ethiopia can be attributed to both inflation and income inequality. Moreover, the study shows a negative long-run relationship between inflation and economic growth. Specifically, a 1-percentage-point increase in the inflation rate would correspond to a 0.54 percentage-point decline in real GDP over the long term (see Table 3). Inflation is one of the largest macroeconomic problems in the universe; therefore, it can reduce economic growth. Inflation can also influence investment decisions. High inflation rates may discourage businesses and investors from making long-term investments because of uncertainty and higher borrowing costs. Reduced investment can hinder economic expansion and, ultimately, affect GDP growth [42].

**Table 3:** The estimated long-run model for lnRGDP.

Variables	lnCPI	lnGINI	constant
Coefficient	-0.549	-0.112	-18.698
t-statistics	(-2.057)	(-5.185)	

Source: Own computation, 2023.

*lnRGDP*_*t*_ = −18.698 − 0.549 *lnCPI*_*t*_ − 0.112 *lnGINI*_*t*_.

Inflation can affect consumer purchasing power. When prices rise, individuals and households may need to spend more money to maintain their standards of living. This can lead to a reduction in real disposable income, reduced consumption, and a slowing down of economic growth. Inflation can impact the labor market and wage dynamics Tanwar [10]. If prices rise faster than wages do, workers may experience a decline in purchasing power. This can lead to reduced morale, lower productivity, and potential labor disputes. Unfavorable labor market conditions can hamper economic growth and limit GDP expansion [43].

Inflation can affect a country’s international competitiveness. If a country experiences higher inflation than its trading partners, its exports may become more expensive than those of other countries, leading to a decrease in export demand. This can harm GDP, especially if a significant portion of the economy relies on exports Chimobi [28]. Inflation can affect the real value of the debt. If prices rise, the real burden of debt decreases because borrowers can repay their loans in less valuable currency. However, lenders may demand higher interest rates to compensate for inflation, which makes borrowing more expensive. These dynamics can impact investment and consumption decisions and consequently influence GDP growth.

This result is similar to that of Geda and Tafere [26], who found that tight monetary and fiscal policies are advised in circumstances where inflation rises over its steady state because it will harm growth. [9,5,44] also found the same result; inflation had negative implications for economic growth.

In the long term, it has been observed that income inequality has a detrimental impact on economic growth. Specifically, when the Gini coefficient, which measures income inequality, increases by 1 percentage point, it leads to a significant decrease of 3.1 percentage points in real economic growth (see Table 3). In the long run, income inequality is generally viewed as a drag on economic growth. When wealth and income are concentrated in the hands of a few individuals or households, they can limit opportunities for others to invest in education, start businesses, or otherwise contribute to economic growth. This can lead to a less dynamic economy, with fewer opportunities for innovation and entrepreneurship.

Income inequality can hinder investment in human capital, such as education and healthcare, particularly for lower-income individuals. This can limit opportunities for skill development and upward mobility, ultimately suppressing overall productivity and long-term GDP growth.

High levels of income inequality can lead to social and political unrest, which affects economic stability and investor confidence. Political instability and social tension can disrupt economic activity, hinder investment, and impede long-term GDP growth [16]. Income inequality can result in unequal access to resources including credit, business opportunities, and quality education. This uneven distribution of resources can hinder entrepreneurship, innovation, and productivity growth, ultimately restraining long-term GDP growth [44]. This finding is similar to [15,31,45,46].

Table 4 suggests that there is a positive relationship between inflation and GDP, which means that a 1-percentage-point increase in real GDP will increase inflation by 0.74 percentage points in the long run. This is known as the Phillips curve, which states that as economic growth (GDP) increases, it leads to higher levels of aggregate demand, which can put upward pressure on prices and contribute to inflationary pressure. In this scenario, sustained economic growth over an extended period could lead to higher inflation rates. The level of economic growth is an important factor. Once the economy is growing rapidly, there may be upward pressure on prices as demand for goods and services increases [26].

**Table 4:** The estimated long-run model for lnCPI.

Variables	lnRGDP	lnGINI	constant
Coefficient	0.74	0.328	9.091
t-statistics	(3.076)	(2.98)	

Source: Own computation, 2023.

*lnCPI*_*t*_ = 9.091 + 0.074 *lnRGD*_*t*_ − 0.328 *lnGINI*_*t*_.

Moreover, real GDP affects inflation through its impact on employment and wages. When real GDP increases, employment opportunities, and wages also increase. This increase in employment and wages can lead to a rise in the claim for goods and services, which can cause an increase in the price level of goods and services, leading to higher price rises.

Conversely, a decrease in the real GDP leads to a decrease in employment opportunities and wages. This decrease in employment and wages can lead to a decrease in the demand for goods and services, which can cause a decrease in the price level of goods and services, leading to lower price increments. This result is supported by those of Dotsey and Sarte [47], Geda and Tafere [26], Makuria [32] and Ayalew [33].

The long-run impact of income inequality on inflation is found to be positive, which means that a 1-percentage-point increase in the GINI coefficient will increase inflation by 0.32 percentage points in the long run (see Table 4). This finding indicates that income inequality can have an important effect on inflation in the long run. When income inequality is high, a small percentage of the population holds a large proportion of wealth. This can lead to an increase in demand for luxury goods and services, which can drive prices up. Additionally, those with higher incomes may be more likely to invest in assets, such as stocks and real estate, which can also contribute to inflation [7].

Furthermore, income inequality can lead to political instability and social unrest, which have economic consequences. For example, protests and strikes can disrupt supply chains and lead to shortages, which can increase prices. In extreme cases, political instability can lead to hyperinflation, as seen in developing countries [19].

Income inequality can also affect the financial system. High-income individuals tend to have a higher propensity to save and invest, which leads to increased financial market activity. This can drive asset price inflation and potentially contribute to broader inflationary pressure in the economy.

The long-run impact of real GDP on income inequality is found to be negative, which means that a 1-percentage-point increase in the real GDP growth rate will decrease income disparity by 0.32 percentage points in the long run (see Table 4). In the long term, GDP growth can lead to a lessening in income inequality. Sustained economic growth can create more opportunities for education and employment, which can help reduce poverty and increase social mobility. Additionally, as economies develop and become more diversified, there may be a shift away from industries dominated by a small number of wealthy individuals or corporations.

Over time, developing economies may undergo structural transformations, moving from being primarily agrarian to industrial or service-oriented economies. This shift can contribute to reducing the income disparities between different sectors and regions. Economic growth allows increased investment in education, training, and skill development. This can lead to better employment opportunities and higher wages for individuals, potentially reducing income inequality over time [14]. Economic growth is often accompanied by technological progress that can create new industries and jobs, promote income mobility, and reduce inequality.

Over time, developing economies may undergo structural transformations, moving from being primarily agrarian to industrial or service-oriented economies. This shift can contribute to reducing the income disparities between different sectors and regions. This finding was supported by [14,15,48,49].

The long-term impact of inflation on income inequality is noteworthy as a 1-percentage-point rise in the inflation rate leads to a 0.561 percentage-point increase in income inequality (see Table 5).

**Table 5:** The estimated long-run model for lnGINI.

Variables	lnRGDP	lnCPI	constant
Coefficient	-0.321	0.562	4.945
t-statistics	(3.076)	(2.632)	

Source: Own computation, 2023.

*lnGINI*_*t*_ = 4.94 − 0.321 *lnRGD*_*t*_ + 0.561 *lnCPI*_*t*_.

When prices rise because of inflation, the purchasing power of money decreases. This can disproportionately affect low-income individuals, who have limited resources to cope with rising costs. If wages do not keep pace with inflation, income inequality may increase as the purchasing power of low-income earners diminishes. Rising inflation can decrease real value transfer; the poor probably cannot protect themselves from rising inflation due to the existence of entry barriers in markets, which can increase income inequality [50].

Inflation can have varying effects on asset types. Typically, assets such as real estate and stocks tend to appreciate during inflationary periods, thus benefiting those who own them. However, lower-income individuals may have limited access to such assets and their wealth may predominantly consist of cash or low-yield savings. As the value of savings erodes with inflation, wealth inequality may widen. One way in which inflation affects income inequality is through its impact on wages. Inflation can lead to an increase in nominal wages, but if the increase in wages is not sufficient to keep up with inflation, then real wages (the purchasing power of wages) will decrease. This means that workers have less money to spend on goods and services, which can lead to a decrease in demand for these goods and services. As a result, businesses may reduce their prices or reduce production, which can lead to job losses and further exacerbate income inequality.

Inflation can also affect income inequality through its impact on savings and investment. When inflation is high, the value of savings and investments decreases over time. This means that people who rely on their savings or investments in income will see a decrease in purchasing power. On the other hand, people with assets that appreciate value during inflationary periods (such as real estate or stocks) may see an increase in their wealth and income, further widening the gap between the rich and the poor. This finding is similar to those of Belay [49], Beck, Demirgüç-Kunt [51], and Albanesi [20].

### Short run analysis

Moderate inflation can have a positive and noteworthy effect on short-term real GDP growth. For instance, a 1-percentage-point rise in the CPI (Consumer Price Index) can lead to a corresponding increase of 0.762 percentage points in real economic growth, as indicated in Table 6. This is because inflation, when kept at a moderate level, can promote spending and investment. Anticipating future price increases, individuals may be inclined to make purchases earlier, thereby bolstering aggregate demand and overall economic activity in the short run [33].

**Table 6:** Short-run model for lnRGDP.

Variables	Error correction	D(lnRGDP(-1))	D(lnCPI(-1))	D(lnGINI(-1))	constant
Coefficient	-0.67	0.478	0.762	-1.672	0.347
t-statistics	(-2.395)	0.876	-3.095	(-3.675)	

Source: Own computation, 2023.

It is important to note that moderate inflation can be considered a sign of a healthy economy, as it indicates a certain level of economic activity. Central banks often aim to maintain the target inflation rate to promote stable economic growth. This finding is supported by [47], who found that inflation can have a positive impact on short-term growth through the mechanism of precautionary savings. During periods of inflation volatility, individuals tend to increase their precautionary savings, which also positively affects growth; Mallik and Chowdhury [52], Ayalew [33] and Ozdemir [11].

In the short term, income inequality has a detrimental effect on real economic growth. It is statistically significant, implying that a 1-percentage-point rise in the Gini coefficient results in a 1.672 percentage-point decrease in real economic growth (see Table 6).

When a small group of individuals possess the majority of income, it can result in a decline in consumer spending overall. This is because individuals with lower incomes tend to spend a larger portion of their earnings, known as a higher marginal propensity to consume. As a result, significant income inequality can lead to a decrease in overall demand, ultimately reducing short-term GDP growth [53].

If incomes of low-income earners remain stagnant or decline, they are likely to allocate a larger portion of their earnings towards goods and services. Consequently, this reduction in consumer spending and aggregate demand has the potential to impede economic growth and potentially result in a recession if the situation persists without intervention [5]. Short-term economic growth can be adversely affected by substantial income inequality. When a considerable portion of the population lacks sufficient purchasing power, it can restrict aggregate demand, resulting in reduced consumer spending and a decline in overall economic activity. This, in turn, can impede short-term GDP growth. These conclusions were substantiated by [2,34,54].

The expected negative sign of the error correction term in the RGDP equation ensures that the actual real GDP can temporarily differ from its long-run equilibrium. Nevertheless, it gradually returns to its equilibrium state. A coefficient of -0.67 for the error correction term indicates that 67 percent of the deviation between the actual real GDP and its equilibrium value is eliminated annually (see Table 6). Therefore, a long-run full adjustment of RGDP would require less than two years.

Real GDP has a positive and significant impact on inflation growth in the short run; that is a 1-percentage-point increase in RGDP will increase real inflation by 0.429 percentage points in the short run (see Table 7). When the economy is operating at its highest capacity or nearing it, an increase in GDP can lead to a greater demand for goods and services. This increase in demand can stimulate price hikes, which eventually lead to inflation. Additionally, during periods of rapid economic expansion, a rise in GDP can temporarily drive prices higher as the demand for goods and services intensifies.

**Table 7:** The estimated short-run model for lnCPI.

Variables	Error correction	D(lnCPI(-1))	D(lnRGDP(-1))	D(lnGINI(-1))	constant
Coefficient	-0.501	0.953	0.429	-1.672	2.098
t-statistics	-2.093	0.365	2.821	-0.931	

Source: Own computation, 2023.

In the immediate term, a rise in real GDP can result in an elevated aggregate demand that surpasses the economy’s capacity to provide goods and services. This can result in demand-pull inflation, where prices rise because of excessive demand. As consumers and businesses spend more, prices may increase to match the increased demand, leading to inflationary pressure. Changes in real GDP can also impact inflation through cost-push factors.

For example, if real GDP growth is accompanied by rising input costs, such as labor or raw materials, businesses may pass these improved costs into consumers in the system of higher prices. This can lead to cost-push inflation, in which rising production costs contribute to overall price increases. This result is similar to [6,9,28]. In the short run, income inequality has an insignificant effect on the inflation-based Table 7.

The expected negative sign of the error correction term in the inflation equation ensures that actual inflation can temporarily deviate from its long-run equilibrium but gradually converges back to that equilibrium. An error correction term of -0.501 implies that 50 percent of the deviation between actual inflation and its equilibrium value is eliminated annually (as shown in Table 7). Consequently, it would take approximately two years for inflation to fully adjust back to its long-run equilibrium.

Real GDP has a positive and significant impact on income inequality in the short run; that is a 1-percentage-point increase in RGDP will increase real inflation by 0.56 percentage points in the short run (see Table 8). In the short term, a rise in GDP can lead to a rise in income inequality. This is because the benefits of economic growth may not be equally distributed among all members of society. For example, those who are already wealthy or who have access to resources such as education and capital may benefit more from economic growth than those who do not [15]. This finding was supported by [34,55–58].

**Table 8:** Estimated short-run model for lnGINI.

Variables	Error correction	D(lnGINI(-1))	D(lnRGDP(-1))	D(lnCPI(-1))	constant
Coefficient	-0.342	0.294	0.562	-0.956	7.512
t-statistics	-3.653	0.256	2.913	-3.769	

Source: Own computation, 2023.

As Inflation increases by a certain level, income inequality also decreases significantly, which means that a 1-percentage-point increase in the inflation rate leads to a decrease in income inequality by 0.956 percentage points in the short run (see Table 8).

Inflation can reduce the real value of debt owed by individuals. In the short run, if inflation outpaces interest rates, borrowers may benefit because their debt burden decreases in real terms. This can be particularly advantageous for lower-income individuals with higher levels of debt. Inflation can lead to wage adjustments in sectors where workers have strong bargaining power or where contracts are linked to inflation. If wages in these sectors increase at a faster rate than the overall inflation rate, income inequality within those sectors [59].

Inflation can stimulate economic growth in the short run, leading to increased demand for labor. This can potentially create more job opportunities and bargaining power for workers, particularly those in lower-income brackets, thereby potentially reducing income inequality. In addition, inflation can result in the redistribution of wealth in favor of individuals or businesses that own assets that appreciate value during inflationary periods. This includes stocks, real estate, and commodities. If wealth redistribution benefits lower-income individuals, it may reduce income inequality to a certain extent. This finding is supported by [60–62].

The estimated coefficient of the VEC term is statistically significant, with a magnitude of -0.34. The negative value of the coefficient indicates that income inequality converges towards long-run equilibrium by approximately 34.2% each year. However, the speed of adjustment towards its long-run equilibrium is moderately slow, as it takes nearly three years to fully adjust in response to shocks introduced in the model.

In this way, Real GDP has a positive and significant impact on income inequality in the short run; that is a 1-percentage-point increase in RGDP will increase real inflation by 0.56 percentage points in the short run. In contrast, the long-run impact of real GDP on income inequality is found to be negative, which means that a 1-percentage-point increase in the real GDP growth rate will decrease income inequality by 0.32 percentage points in the long run. Ethiopia is one of the least developed countries. Based on this result, in the initial stages, real GDP has a positive implication on income inequality; however, over time growth in real GDP hurt income inequality.

### Granger causality test result

A simple linear regression model, typically employed in a bivariate system, is a direct method for examining the cause-and-effect relationship between variables. This approach, known as a dynamic relationship, seeks to understand the underlying dynamic causality. However, the simple linear regression model has its limitations in capturing this dynamic causality between variables. [36] The Granger causality test was introduced to analyze the dynamic causality between variables efficiently. To explore the dynamic relationships between variables, we employed the Granger causality test using a VECM model. Its purpose is to ascertain if changes in one variable have a causal impact on changes in another variable. Logical consistency in the analysis of the Granger causality test is crucial for determining the direction of causation between the two variables. In the case of Ethiopia, we present the results of the Granger causality test regarding the connection among inflation, economic growth, and income inequality in Table 9.

**Table 9:** Pair-wise granger causality test.

Null hypothesis	Observation	F-Statistic	Probability
LnGINI does not Granger Cause LNRGDP	42	10.6832	0.0023
LNRGDP does not Granger Cause LnGINI		13.1659	0.0009
LnCPI does not Granger Cause LNRGDP	42	5.0828	0.0302
LNRGDP does not Granger Cause LnCPI		4.6492	0.0376
LnCPI does not Granger Cause LnGINI	42	16.4018	0.0029
LnGINI does not Granger Cause LnCPI		0.5679	0.4559

Source: Own computation, 2023.

As can be seen from Table 9, we reject the null hypothesis that LnGINI does not Granger-cause LNRGDP and the null hypothesis that LNRGDP does not Granger-cause LnGINI. Therefore, the Granger causality runs in two ways from LNGDP to LNENU and vice versa; therefore, the Granger causality between inflation and economic growth is bidirectional. In addition, we reject the null hypothesis that LnCPI does not Granger-cause LNRGDP and the null hypothesis that LNRGDP does not Granger-cause LnCPI. This means that bidirectional Granger causality exists between real GDP and inflation. However, we failed to reject the null hypothesis that LnGINI does not Granger-cause LnCPI and only rejected the null hypothesis that LnCPI does not Granger-cause LnGINI. This implies unidirectional Granger causality from inflation to real income inequality.

## Conclusion and policy recommendations

### Conclusion

Currently, there is an indisputable debate among economic researchers and policymakers on the long-run and short-run dynamics between inflation, income inequality, and economic growth. Therefore, this study analyzes the nexus between inflation, income inequality, and economic growth in Ethiopia, utilizing a co-integrated Vector Autoregression (VAR) model from the year 1980–2022, provides valuable insights into the interplay among these variables and also has important implications for policymakers and stakeholders in understanding the complex dynamics of these variables. Johansen cointegrated was also employed to determine the existence of long-run relationships between the variables. The Johansen cointegrated test confirmed the existence of three cointegrated relationships between the dependent and independent variables, based on λmax and λtrace test statistic values. In the long run, the model estimation shows that both inflation and income inequality have negative effects on real GDP. Inflation is affected by inflation and real GDP in the long run. Income inequality has a positive and statistically significant relationship with real GDP and inflation in the long run.

Moreover, in the near term, there is a positive and substantial correlation between real GDP and inflation, while real GDP exhibits a negative and statistically noteworthy association with income inequality. Additionally, the income inequality model for the short run demonstrates that both real GDP and inflation have a negative and significant relationship with income inequality. However, only real GDP demonstrates a positive and significant connection with inflation in the short term. The short-run adjustment coefficient for all three variables was significant, signifying that all three variables will converge to long-run equilibrium in the future. Results from the Granger causality test indicate bidirectional causality between real GDP and inflation, as well as real GDP and income inequality. However, the Granger causality test demonstrates unidirectional causality from inflation to income inequality.

### Policy recommendations

Given the significant long- and short-run relationship between inflation, income inequality, and economic growth, the following policy recommendations emanate from this study: strong government policies regarding taxation and social safety net policy, which could significantly shape the impact of inflation on income inequality. Applying progressive tax policies and well-designed social safety nets. Redistribution policies: Governments may choose to increase social welfare spending, redistribution policies, adjust tax policies, or apply progressive tax; implement targeted redistribution programs such as welfare programs, social safety nets, or minimum wage to mitigate the impact of inflation on low-income individuals. Implementing a robust inflation-targeting framework to maintain price stability. The central bank should set an inflation target consistent with long-term economic growth, and establish effective monetary policy tools to manage inflation. Close coordination among monetary authorities, fiscal policymakers, and other relevant institutions is essential for achieving inflation targets and ensuring macroeconomic stability. Develop and implement comprehensive policies aimed at reducing income inequality, such as progressive taxation, targeted social protection programs, and labor market reforms. The main objective is to enhance the availability of top-notch education, healthcare, and vital services to foster equal opportunities among every individual. Adopt policies that foster sustainable and inclusive economic growth. Invest in infrastructure development, particularly in rural areas, to enhance productivity and connect remote regions to markets. Encourage private sector participation through investment-friendly policies, streamlined regulations, and improved access to finance. Promote entrepreneurship and innovation to create employment opportunities, particularly for youths and women. Encouraging small and medium enterprises (SMEs) and entrepreneurship can also contribute to inclusive growth. Policies should aim to achieve macroeconomic stability by balancing inflation and GDP growth. Central banks and fiscal authorities must coordinate their actions to ensure that inflation is controlled while supporting robust GDP growth. A balanced approach is crucial for avoiding extreme fluctuations in inflation or GDP. Central banks should have the autonomy and tools necessary to conduct an effective monetary policy, such as adjusting interest rates and managing money supply appropriately, responding flexibly to changing economic conditions, and being transparent in communicating their policy decisions. Governments must implement responsible fiscal policies that support sustainable economic growth while considering inflationary pressures. This involves maintaining a prudent balance among government spending, taxation, and borrowing. Fiscal policies should be geared towards long-term stability, ensuring that government finances are sustainable and supportive of private-sector investments. Structural reforms play a vital role in promoting long-term economic growth and mitigating the impact of inflation on the GDP. These reforms may include measures to enhance labor market flexibility, promote competition, improve education and skills training, streamline regulations, and encourage innovation and entrepreneurship. By improving productivity and reducing supply-side constraints, structural reforms can support sustainable GDP growth, while reducing inflationary pressure. Foster fair labor practices, protect workers’ rights, and promote collective bargaining. Strengthen labor market institutions and ensure adequate social protection for workers, including fair wages, reasonable working hours, and safe working conditions. These reforms have contributed to reducing income disparities and enhancing productivity and economic growth., and good governance in the public and private sectors. Strengthen institutions responsible for combating corruption and promoting fair competition. This study was analyzed only the interaction between inflation, income inequality and economic growth till 2022, it does not consider other confounding factors that could be influenced their causality. Therefore, future research should be done on the nexus between inflation, income inequality and economic growth beyond 2022 by considering other cofounding factors using another econometrics model.

## Supporting information

S1 Dataset (DTA)
